# Phase II Study of Personalized Peptide Vaccination with Both a Hepatitis C Virus-Derived Peptide and Peptides from Tumor-Associated Antigens for the Treatment of HCV-Positive Advanced Hepatocellular Carcinoma Patients

**DOI:** 10.1155/2015/473909

**Published:** 2015-10-11

**Authors:** Shigeru Yutani, Kazuomi Ueshima, Kazumichi Abe, Atsushi Ishiguro, Junichi Eguchi, Satoko Matsueda, Nobukazu Komatsu, Shigeki Shichijo, Akira Yamada, Kyogo Itoh, Tetsuro Sasada, Masatoshi Kudo, Masanori Noguchi

**Affiliations:** ^1^Cancer Vaccine Center, Kurume University, Kurume 839-0863, Japan; ^2^Department of Gastroenterology and Hepatology, Kinki University Faculty of Medicine, Osaka 589-8511, Japan; ^3^Department of Digestive, Rheumatism and Collagen Internal Medicine, Fukushima Prefectural Medical College, Fukushima 960-1295, Japan; ^4^Department of Medical Oncology, Hirosaki University Graduate School of Medicine, Hirosaki 036-8562, Japan; ^5^Department of Gastroenterology, Showa University School of Medicine, Tokyo 142-8555, Japan; ^6^Department of Immunology, Kurume University School of Medicine, Kurume 830-0011, Japan; ^7^Research Center for Innovative Cancer Therapy, Kurume University, Kurume 830-0011, Japan

## Abstract

*Objective*. To evaluate safety and immune responses of personalized peptide vaccination (PPV) for hepatitis C virus- (HCV-) positive advanced hepatocellular carcinoma (HCC). *Patients and Methods*. Patients diagnosed with HCV-positive advanced HCC were eligible for this study. A maximum of four HLA-matched peptides were selected based on the preexisting IgG responses specific to 32 different peptides, which consisted of a single HCV-derived peptide at core protein positions 35–44 (C-35) and 31 peptides derived from 15 different tumor-associated antigens (TAAs), followed by subcutaneous administration once per week for 8 weeks. Peptide-specific cytotoxic T lymphocyte (CTL) and IgG responses were measured before and after vaccination. *Results*. Forty-two patients were enrolled. Grade 3 injection site skin reaction was observed in 2 patients, but no other PPV-related severe adverse events were noted. Peptide-specific CTL responses before vaccination were observed in only 3 of 42 patients, but they became detectable in 23 of 36 patients tested after vaccination. Peptide-specific IgG responses were also boosted in 19 of 36 patients. Peptide-specific IgG1 responses to both C-35 and TAA-derived peptides could be potentially prognostic for overall survival. *Conclusion*. Further clinical study of PPV would be warranted for HCV-positive advanced HCC, based on the safety and strong immune induction.

## 1. Introduction

Although sorafenib has been approved for advanced hepatocellular carcinoma (HCC), which is defined as metastatic or locally advanced disease not amenable to locoregional therapies, the efficacy of this agent was modest and the median survival time (MST) was around 10 months [1, 2]. In addition, no other systemic treatments have shown obvious efficacy in the past 5 years [3, 4]. Nevertheless, new approaches to immunotherapy, such as glypican-3 targeting peptide vaccine and anti-CTLA4 treatment, have shown promising results in the early phase of clinical studies [5–9].

We have developed a novel regimen of personalized peptide vaccination (PPV) that can be used to treat cancer patients with many different HLA-class I types. In this approach, the preexisting host immunity is analyzed to select 4 peptides from among 31 pooled peptides derived from 15 different TAAs, which are then administered as a vaccination [10–15]. PPV has the potential to prolong overall survival (OS) in advanced cancer patients who fail to respond to standard chemotherapy. We also reported a prophylactic effect of PPV with hepatitis C virus- (HCV-) derived peptides against the development of HCC associated with HCV [16–18]. The HCV-core peptide at positions 35–44 (C-35 peptide), which can induce cytotoxic T lymphocyte (CTL) activity in many different HLA-class I types, is a key peptide in the prophylactic effect [19]. In the current study, therefore, we conducted a phase II study of PPV, in which 4 peptides were selected from among 32 different peptides that consisted of a C-35 peptide and 31 peptides derived from 15 TAAs, for HCV-positive advanced HCC patients in order to evaluate the safety and immune responses.

## 2. Patients and Methods

### 2.1. Patients

Patients who were diagnosed with HCV-positive advanced HCC as defined by metastatic or locally advanced disease and were not candidates for locoregional therapies were eligible for this study. Staging was carried out according to the Japanese integrated staging system (Liver Cancer Study Group of Japan) [8, 20]. The patients had to show positive IgG responses to at least 2 of the 32 different vaccine candidate peptides, as reported previously [10–18]. Other inclusion criteria were as follows: age between 20 and 80 years; an Eastern Cooperative Oncology Group (ECOG) performance status of 0 or 1 at the time of first visit; positive status for the human leukocyte antigens (HLA) A2, A24, A3 supertype (A3, A11, A31, or A33), or A26; life expectancy of at least 12 weeks; and adequate hematologic, hepatic, and renal function. Exclusion criteria included pulmonary, cardiac, or other systemic diseases; an acute infection; a history of severe allergic reactions; pregnancy or nursing; and other inappropriate conditions for enrollment as judged by clinicians. The protocol was approved by the Ethical Committee of each university and registered in the UMIN Clinical Trials Registry (UMIN000003520, UMIN000005634). All patients were given a full explanation of the protocol and provided their informed consent before enrollment.

### 2.2. Clinical Protocol

This was a phase II study conducted by Kurume University, Kinki University, Hirosaki University, Fukushima Prefectural College, and Showa University Hospitals. Primary endpoint was to evaluate the safety and immunological responses. Secondary endpoint was to evaluate a clinical benefit from the viewpoint of OS. C-35 peptide (YLLPRRGPRL) derived from the HCV core protein, which was applicable for all the above-listed HLA types as reported previously [16–19], and 31 peptides, which were derived from 15 different TAAs [12 peptides for HLA-A2, 16 peptides for HLA-A24, 9 peptides for HLA-A3 supertypes (-A3, -A11, -A31, and -A33), and 4 peptides for HLA-A26] (Supplementary Table 1, Supplementary Material available online at http://dx.doi.org/10.1155/2015/473909), were employed for vaccination. These peptides were prepared under the conditions of Good Manufacturing Practice by the Polypeptide Laboratories (San Diego, CA) and American Peptide Company (Vista, CA).

Two to four peptides for vaccination to individual patients were selected in consideration of the HLA typing and preexisting host immunity, as assessed by the titers of IgG specific to each of the 32 different vaccine candidates before vaccination [10–18]. The selected peptides were subcutaneously administered with incomplete Freund's adjuvant (Montanide ISA-51; Seppic, Paris, France) once a week for 8 consecutive weeks. During the PPV, only best supportive care was allowed except for patients who were receiving chemotherapy or targeted therapy at the time of entry. Tumor markers (TM), *α*-fetoprotein (AFP), and des-*γ*-carboxy prothrombin (DCP) were measured before and after the 8th vaccination. Adverse events were monitored according to the National Cancer Institute Common Terminology Criteria for Adverse Events version 4.0 (NCI-CTC Ver. 4.0).

### 2.3. Measurement of IgG and CTL Responses

Humoral immune responses specific to each of the 32 peptide candidates were determined by measuring the levels of peptide-specific IgG and IgG subclasses (IgG1, IgG2, IgG3, and IgG4) using the Luminex system (Luminex, Austin, TX), as previously reported [10–15]. If the titers of peptide-specific IgG to at least one of the vaccinated peptides after the 8th vaccination were more than twofold higher than those before vaccination, the changes were considered to be significant, as previously reported [10–15]. CTL responses specific to the vaccinated peptides were evaluated by interferon- (IFN-) *γ* ELISPOT assay using peripheral blood mononuclear cells (PBMCs) before and after vaccination as previously reported [10–15]. As a control, CTL responses specific to CEF peptides (MABTECH, Cincinnati, OH), a mixture of virus-derived CTL epitopes, were also examined.

### 2.4. Statistical Analyses

OS was calculated from the first day of peptide vaccination until the date of death or the last date when the patient was known to be alive. The survival analysis was performed with the Kaplan-Meier method, and a comparison of the survival curves was performed with the log-rank test or Wilcoxon test. Spearman's correlation index was utilized to examine the association among the values of IgG and IgG subclasses. Values of *P* < 0.05 were considered to indicate statistical significance. All statistical analyses were conducted using the JMP software package, version 10 (SAS Institute Inc., Cary, NC).

## 3. Results

### 3.1. Patients' Characteristics

Between December 2000 and May 2013, 42 patients with HCV-positive advanced HCC (Stage IVa: 15 patients; Stage IVb: 27 patients) were enrolled in this study (Table 1). The Japanese integrated staging (JIS) scores [20, 21] of the 42 patients were 3 (*n* = 21), 4 (*n* = 18), and 5 (*n* = 3). Previously conducted regimens of locoregional therapies included hepatectomy (*n* = 14), surgery other than hepatectomy (*n* = 2), radiation (*n* = 9), transcatheter arterial embolization (TAE) (*n* = 23), transcatheter arterial chemoembolization (TACE) (*n* = 16), hepatic arterial infusion chemotherapy (HAIC) (*n* = 16), radiofrequency ablation (RFA) (*n* = 15), percutaneous ethanol injection therapy (PEIT) (*n* = 6), and microwave coagulation therapy (MCT) (*n* = 3). The median number of these treatment regimens was 2, with a range of 0 to 5. Previously conducted systemic therapies for advanced HCC were sorafenib (*n* = 21), 5-FU based drugs (*n* = 6), and new clinical trials (*n* = 9), with a median regimen number of 1 and a range of 0 to 4.

The median value of AFP at the time of the first visit, two weeks before the 1st vaccination, was 376 ng/mL (3.7 to 103,000 ng/mL), while the median value of DCP was 2,335 mAU/mL (11 to 778,000 mAU/mL). Thirty-six patients received 8 vaccinations and completed the protocol, whereas the remaining 6 patients dropped from the protocol before the 8th vaccination due to rapid disease progression (*n* = 5) or of their own will (*n* = 1). The median number of peptide vaccinations was 8, with a range of 3 to 8. Thirty patients received PPV alone, 10 patients received PPV with sorafenib, 1 patient received PPV with S-1, and 1 patient received PPV with HAIC.

### 3.2. Adverse Events

Skin reactions of grades 1, 2, and 3 at the injection sites were observed in 15, 4, and 2 patients, respectively, but no other PPV-related severe adverse events were observed (Table 2). Fourteen grade 3 adverse events were observed during vaccination, with 10 events occurring in patients treated with PPV alone and 4 events occurring in those with PPV and combined therapies. No grade 4 adverse events were observed, whereas a grade 5 adverse event was observed in 1 patient with PPV and sorafenib (pleural infection). All of them except for skin reaction at injection sites were considered to be due to disease progression or combined therapies judged by an independent ethical committee.

### 3.3. Immune Responses

Both peptide-specific CTL and IgG responses were analyzed in prevaccination blood samples from all 42 patients and in postvaccination samples from 36 patients who completed the 8th vaccination. CTL responses to the vaccinated peptides were detectable in only 3 of 42 patients before vaccination (2 patients for C-35 peptide and 1 patient for TAA peptide) (Supplementary Table 2). However, it became detectable after vaccination in 23 of 36 patients: CTL responses specific to the C-35 peptide were observed in 19 of 36 patients tested, and those specific to the TAA-derived peptides were observed in 15 of 36 patients. We also tested CTL responses to CEF peptides, a mixture of virus-derived CTL epitopes, as a control. They were present in 15 of 42 patients before vaccination and in 19 of 36 patients after the 8th vaccination (Supplementary Table 2). Increase or decrease of CTL responses to CEF peptides was observed in 15 or 5 of 36 patients, respectively.

Peptide-specific IgG responses before vaccination were observed in all patients, with very high levels of IgG titers to the C-35 peptide in most of them. Augmentation of the IgG responses to at least one of the vaccinated peptides after vaccination was observed in 19 of 36 patients tested, with an increase of IgG specific to the C-35 peptide in 5 of 36 patients and an increase of IgG specific to TAA-derived peptides in 19 of 36 patients (Supplementary Table 2). To better understand humoral immune responses, the levels of IgG subclasses (IgG1, IgG2, IgG3, and IgG4) specific to the vaccinated peptides before and after vaccination were also measured (Supplementary Table 3). There was a significant correlation between peptide-specific IgG and IgG1 (Spearman rank correlation coefficient = 0.865), but not between IgG and IgG2, IgG3, or IgG4 (Spearman rank correlation coefficient: IgG versus IgG2 = 0.376, IgG versus IgG3 = 0.371, and IgG versus IgG4 = 0.310). In contrast, there were substantial correlations among peptide-specific IgG2, IgG3, and IgG4 (Spearman rank correlation coefficient: IgG2 versus IgG3 = 0.554, IgG2 versus IgG4 = 0.491, and IgG3 versus IgG4 = 0.556).

### 3.4. Clinical Responses

**Figure 1:**
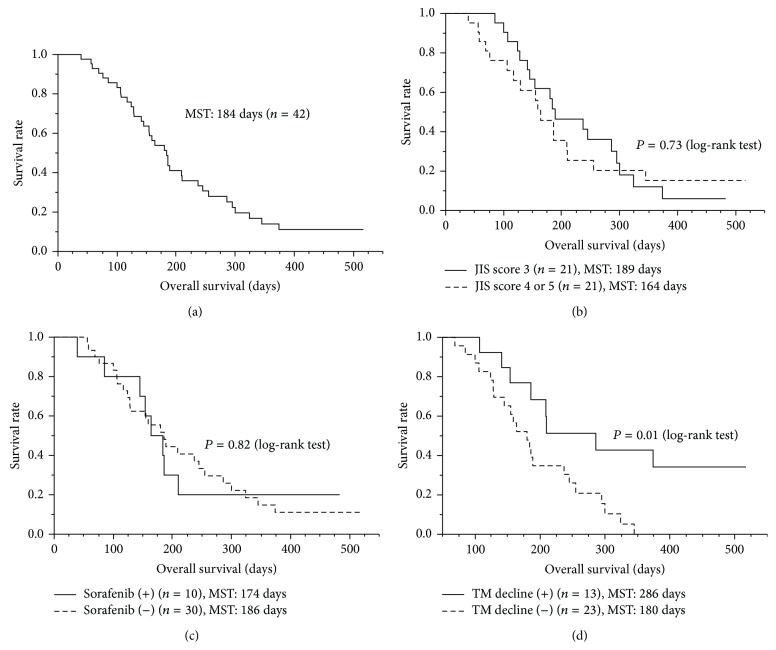
Survival analysis. The survival analysis was performed with the Kaplan-Meier method, and a comparison of the survival curves was performed with the log-rank test. (a) The median survival time (MST) from the first vaccination of PPV was 184 days in 42 patients. (b) The patients with a JIS score of 3 (MST, 189 days) did not show significantly different survival, compared to those with a JIS score of 4 or 5 (MST, 164 days) (*P* = 0.73). (c) Combination therapy with sorafenib did not affect OS (*P* = 0.82). (d) Patients with decreased TM (tumor markers), AFP, or DCP, after vaccination (MST, 286 days) showed longer survival than those without it (MST, 180 days) (*P* = 0.01).

AFP was decreased after vaccination in 9 of 33 patients, who showed abnormal elevation of serum AFP (>10 ng/mL) before vaccination (Supplementary Table 2). Decrease in another tumor marker, DCP, after vaccination was also observed in 9 of 33 patients, who showed abnormal elevation of serum DCP (>40 mAU/mL) before vaccination (Supplementary Table 2). The MST of the 42 patients was 184 days (Figure 1(a)). It is of note that the MST of patients with a JIS score of 3 (189 days) was not substantially different from that of patients with a JIS score of 4 or 5 (164 days) (Figure 1(b), *P* = 0.73 by log-rank test). In addition, combination therapy with sorafenib had no effect on the MST (Figure 1(c), *P* = 0.82 by log-rank test). As expected, however, patients showing decrease in AFP or DCP after the 8th vaccination (*n* = 13; MST, 286 days) showed longer survival than those without such decreases (*n* = 23; MST, 180 days) (Figure 1(d); *P* = 0.01 by log-rank test, *P* = 0.046 by Wilcoxon test).

**Figure 2:**
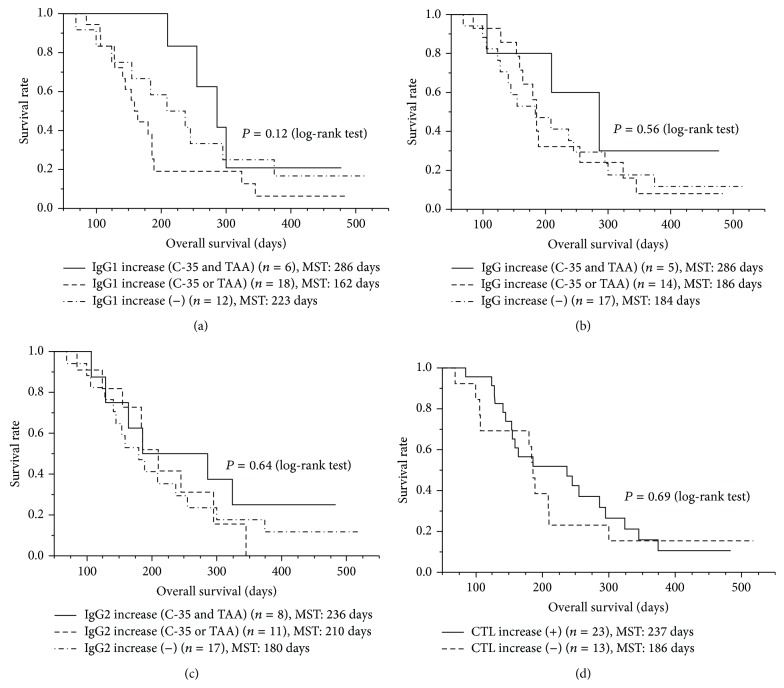
Immune response and overall survival. Association between immune responses and OS was examined with the Kaplan-Meier method, and a comparison of the survival curves was performed with the log-rank test. (a) The peptide-specific IgG1 response was potentially prognostic (*P* = 0.12 by log-rank test, *P* = 0.06 by Wilcoxon test); all 6 patients showing increased IgG1 responses to both the C-35 peptide and TAA-derived peptides survived more than 210 days, and their MST (286 days) tended to be longer than that of patients showing increased IgG1 responses to either peptide (162 days) or that of patients showing no increase to any peptide (223 days). (b) The peptide-specific IgG response was not prognostic (*P* = 0.56). (c) The peptide-specific IgG2 response was not prognostic (*P* = 0.64). (d) The peptide-specific CTL response was not prognostic (*P* = 0.69).

Notably, all 6 patients showing increased IgG1 responses to both C-35 peptide and TAA-derived peptides survived more than 210 days, and their MST (286 days) tended to be longer than that of patients showing an increased IgG1 response to either peptide (*n* = 18, 162 days) or that of patients showing no increase to any peptide (*n* = 12, 223 days) (Figure 2(a); *P* = 0.12 by log-rank test, *P* = 0.06 by Wilcoxon test). However, peptide-specific IgG (Figure 2(b); *P* = 0.56 by log-rank test), IgG2 (Figure 2(c), *P* = 0.64 by log-rank test), IgG3, or IgG4 responses (data not shown) as well as peptide-specific CTL responses (Figure 2(d), *P* = 0.69 by log-rank test) did not show prognostic significance.

## 4. Discussion

The tumor immunity against HCV-positive advanced HCC was reported to be deeply suppressed [22]. For example, molecules involved in T cell check points have been suggested to inhibit CTL responses against tumor cells in advanced HCC [9]. As expected, the current study demonstrated that CTL responses to the vaccinated peptides, but not to virus-derived peptides, before vaccination were rarely observed, indicating that the antitumor immunity in the enrolled patients was severely depressed. However, CTL responses to the vaccinated peptides became detectable at the end of the 8th vaccination in 23 of 36 patients tested. In addition, PPV did not suppress but rather increased the CTL responses to virus-derived peptides. The peptide-specific IgG responses were also boosted in 19 of 36 patients tested. Severe PPV-related adverse events were rarely observed, in agreement with our previous reports [10–18]. In sum, these results indicate that PPV might be a useful approach for HCV-positive advanced HCC patients, who fail to respond to various locoregional and/or systemic treatment regimens, from the viewpoint of both safety and immunological responses.

The MST of the enrolled patients from the first vaccination of PPV was 184 days, with 189 days for the patients with JIS score of 3 and 164 days for those with JIS score of 4 or 5. The current data might be promising for the patients with the JIS score of 4 or 5, since the MST of these patients was reported to be around 3 to 4 months [20, 21]. The MSTs of patients treated with PPV alone or PPV in combination with sorafenib were 186 or 174 days, respectively. No grade 4 or 5 adverse events were observed in patients with PPV alone, whereas a grade 5 adverse event (pleural infection) was observed in a patient receiving PPV and sorafenib. These results suggested that the combination of sorafenib and PPV had no additive benefit, although the scale of the study was small.

From the viewpoint of biomarkers, the peptide-specific IgG1 response was suggested to be a potentially prognostic factor in this study, since all 6 patients showing boosted IgG1 responses to both C-35 peptide and TAA-derived peptides survived more than 210 days, and their MST (286 days) tended to be longer than that of patients showing boosted IgG1 responses to either peptide alone (162 days) or that of patients showing no increase in response to any peptide (223 days) (*P* = 0.12 by log-rank test, *P* = 0.06 by Wilcoxon test). In contrast, the peptide-specific IgG2 response did not show prognostic significance. Since IgG1, but not IgG2, is known to enhance antibody-mediated opsonization and phagocytosis of antigens, peptide-specific IgG1 may enhance antitumor immunity through phagocytosis and cross-presentation of antigen peptides [23]. Further studies will be needed to clarify the mechanisms.

In contrast to IgG1 responses as a potential prognostic biomarker, the peptide-specific CTL response was not well correlated with OS in these patients under PPV. This may have been mainly due to the small size of patient numbers. Indeed, we suggested that the peptide-specific IgG response was more useful than the peptide-specific CTL response as a prognostic biomarker for patients under PPV, primarily because monitoring of IgG responses shows higher sensitivity than that of CTL responses [24].

## 5. Conclusion

The current study indicated that PPV with both a HCV-derived CTL epitope peptide and 31 peptides from TAAs could be recommended for the next step of a clinical trial in HCV-positive advanced HCC patients, because of safety and strong immune induction.

## Supplementary Material

The supplementary material provides the list of peptides derived from tumor-associated antigens that were selected and administered for PPV (Supplementary Table 1), detailed information (IgG or CTL responses to the vaccines, tumor markers, and overall survival) before and after vaccination in each patient (Supplementary Table 2), and detailed humoral immune (IgG1, IgG2, IgG3, and IgG4) responses to the vaccines before and after vaccination in each patient (Supplementary Table 3).

## Figures and Tables

**Table 1:** Patients' characteristics (*n* = 42).

Factor	Number
Age	
Median (range)	70 (48–80)
Gender	
Male	34
Female	8
ECOG performance status	
0	32
1	10
HLA type	
A24	24
A2	21
A3 supertype	14
A26	13
Clinical stage	
IVa	15
IVb	27
JIS score	
3	21
4	18
5	3
Previously conducted treatments	
Locoregional	
Hepatectomy	14
Surgery other than hepatectomy	2
Radiation	9
Transcatheter arterial embolization (TAE)	23
Transcatheter arterial chemoembolization (TACE)	16
Hepatic arterial infusion chemotherapy (HAIC)	16
Radiofrequency ablation (RFA)	15
Percutaneous ethanol injection therapy (PEIT)	6
Microwave coagulation therapy (MCT)	3
Systemic	
Sorafenib	21
5-FU based chemotherapies	6
Other clinical trials	9
AFP at first visit	
Median (range), ng/mL	376 (3.7–103000)
DCP at first visit	
Median (range), mAU/mL	2335 (11–778000)
Number of vaccinations	
Median (range)	8 (3–8)
Combination therapy	
None	30
Sorafenib	10
Chemotherapy	2

ECOG: Eastern Cooperative Oncology Group; JIS: Japanese integrated staging; AFP: *α*-fetoprotein; DCP: des-*γ*-carboxy prothrombin.

**Table 2:** Adverse events during the PPV (*n* = 42).

Event	Number					
	Grade 1	Grade 2	Grade 3	Grade 4	Grade 5	Total (%)
Injection site skin reaction	15	4	2	0	0	21 (50%)
Blood/bone marrow						
Anemia	7	4	0	0	0	11 (26%)
Lymphopenia	9	1	0	0	0	10 (24%)
Neutropenia	0	2	0	0	0	2 (5%)
Thrombocytopenia	7	0	0	0	0	7 (17%)
Leukopenia	3	1	0	0	0	4 (10%)
Laboratory						
AST increase	4	6	4	0	0	14 (33%)
ALT increase	10	1	2	0	0	13 (31%)
ALP increase	9	2	0	0	0	11 (26%)
GGT increase	7	3	2	0	0	12 (29%)
Bilirubin increase	2	2	0	0	0	4 (10%)
Creatinine increase	2	1	0	0	0	3 (7%)
Gastrointestinal disorders						
Anorexia	5	3	0	0	0	8 (19%)
Abdominal distension	2	0	0	0	0	2 (5%)
Ascites	2	1	1	0	0	4 (10%)
Constipation	0	2	0	0	0	2 (5%)
Edema limbs	2	0	0	0	0	2 (5%)
Fever	5	0	0	0	0	5 (12%)
Malaise	3	0	0	0	0	3 (7%)
Pain	1	3	2	0	0	6 (14%)
Pruritus	2	0	0	0	0	2 (5%)
Eruption	2	1	0	0	0	3 (7%)
Urinary incontinence	0	1	0	0	0	1 (2%)
Pleural infection	0	0	0	0	1	1 (2%)
Hypertension	0	0	1	0	0	1 (2%)
Insomnia	0	1	0	0	0	1 (2%)
