# Supplementary material for: Phase II Study of Personalized Peptide Vaccination with Both a Hepatitis C Virus-Derived Peptide and Peptides from Tumor-Associated Antigens for the Treatment of HCV-Positive Advanced Hepatocellular Carcinoma Patients
Source: J Immunol Res. 2015 Oct 11;2015:473909. doi: 10.1155/2015/473909 (PMC4619935; doi:10.1155/2015/473909)
Supplement: Supplementary file 1 — The supplementary material provides the list of peptides derived from tumor-associated antigens that were selected and administered for PPV (Supplementary Table 1), detailed information (IgG or CTL responses to the vaccines, tumor markers, and overall survival) before and after vaccination in each patient (Supplementary Table 2), and detailed humoral immune (IgG1, IgG2, IgG3, and IgG4) responses to the vaccines before and after vaccination in each patient (Supplementary Table 3). [file 473909.f1.pdf]

Supplementary Table 1. Peptides from tumor-associated antigens

Symbol for peptide	Protein	Position of peptide	Amino acid sequence	HLA type
CypB-129	Cyclophilin B	129-138	KLKHYGPGWV	A2, A3sup[a]
Lck-246	p56Lck	246-254	KLVERLGAA	A2
Lck-422	p56Lck	422-430	DVWSFGILL	A2, A3sup
MAP-432	ppMAPkkk	432-440	DLLSHAFFA	A2, A26
WHSC2-103	WHSC2	103-111	ASLDSDPWV	A2, A3sup, A26
HNRPL-501	HNRPL	501-510	NVLHFFNAPL	A2, A26
UBE-43	UBE2V	43-51	RLQEWCSVI	A2
UBE-85	UBE2V	85-93	LIADFLSGL	A2
WHSC2-141	WHSC2	141-149	ILGELREKV	A2
HNRPL-140	HNRPL	140-148	ALVEFEDVL	A2
SART3-302	SART3	302-310	LLQAEAPRL	A2
SART3-309	SART3	309-317	RLAEYQAYI	A2
SART2-93	SART2	93-101	DYSARWNEI	A24
SART3-109	SART3	109-118	VYDYNCHVDL	A24, A3sup, A26
Lck-208	p56Lck	208-216	HYTNASDGL	A24
PAP-213	PAP	213-221	LYCESVHNF	A24
PSA-248	PSA	248-257	HYRKWIKDTI	A24
EGFR-800	EGF-R	800-809	DYVREHKDNI	A24
MRP3-503	MRP3	503-511	LYAWEPSFL	A24
MRP3-1293	MRP3	1293-1302	NYSVRYRPGL	A24
SART2-161	SART2	161-169	AYDFLYNYL	A24
Lck-486	p56Lck	486-494	TFDYLRSVL	A24
Lck-488	p56Lck	488-497	DYLRSVLEDF	A24
PSMA-624	PSMA	624-632	TYSVSFDSL	A24
EZH2-735	EZH2	735-743	KYVGIEREM	A24
PTHrP-102	PTHrP	102-111	RYLTQETNKV	A24
SART3-511	SART3	511-519	WLEYYNLER	A3sup
SART3-734	SART3	734-742	QIRPIFSNR	A3sup
Lck-90	p56Lck	90-99	ILEQSGEWWK	A3sup
Lck-449	p56Lck	449-458	VIQNLERGYR	A3sup
PAP-248	PAP	248-257	GIHKQKEKSR	A3sup

[a]A3sup; HLA-A3 supertype (A3, A11, A31, and A33)

Supplementary Table 2. Immune responses, tumor markers, and overall survival

Patient No.	Peptide[a]	IgG response[b]		CTL response[c]		CTL response to CEF[c]		AFP[d]		DCP[d]		OS (days)
		Before	After	Before	After	Before	After	Before	After	Before	After	
1	C-35	7250	7768	0	679	0	0	3479	671	874	583	141
	Lck-208	98	0	0	0							
	PAP-213	176	0	0	0							
	PTHrP-102	84	0	0	1311							
2	C-35	16547	14532	0	130	0	0	774	901	1820	1820	295
	Lck-208	12	0	0	0							
	PAP-213	25	18	0	0							
	PTHrP-102	33	0	0	0							
3	C-35	8959	22289	0	0	0	0	49	17	15300	20800	107
	Lck-246	214	6989	0	0							
	CypB-129	153	171	0	0							
	WHSC2-141	22	20	0	0							
4	C-35	44	106	0	0	0	0	13300	15700	20008	5326	210
	Lck-208	37	16	0	0							
	PSMA-624	30	73	0	0							
	PAP-213	26	711	0	0							
5	C-35	19100	19828	0	1407	899	1477	238	409	2600	49800	124
	CypB-129	330	315	0	0							
	UBE2V-43	39	55	0	698							
	HNRPL-140	270	263	0	0							
6	C-35	14375	NA	0	NA	0	NA	2688	NA	7861	NA	76
	HNRPL-501	713	NA	0	NA							
	ppMAPkkk-432	385	NA	0	NA							
	WHSC2-103	98	NA	0	NA							
7	C-35	3413	3208	0	0	3063	4658	5480	6130	40900	45400	106
	MRP3-1293	259	270	0	0							
	Lck-488	272	289	0	0							
	SART2-93	173	278	0	0							
8	C-35	22945	24550	0	803	0	0	151	320	384	4055	159
	SART2-93	148	153	0	0							
	Lck-488	135	3924	0	79							

	MRP3-1293	129	148	0	0							
9	C-35	10060	19651	0	0							
	UBE2V-85	7035	15664	0	0	0	0	11190	25150	24474	43932	186
	Lck-422	6165	6160	0	0							
	UBE2V-43	4421	41049	0	0							
10	C-35	452	3286	0	39							
	PAP-213	452	33115	0	1015	2963	1067	53	18	148	75	477[e]
	Lck-486	148	25639	0	0							
	Lck-488	158	572	0	1353							
11	C-35	24785	NA	0	NA							
	WHSC2-103	1787	NA	0	NA	0	NA	4	NA	53	NA	39
	ppMAPkkk-432	394	NA	0	NA							
	WHSC2-141	130	NA	0	NA							
12	C-35	13348	15630	0	0							
	SART3-109	2742	2002	0	0	324	0	4986	5058	330	1474	180
	Lck-90	160	181	0	0							
	HNRPL-501	154	959	0	0							
13	C-35	8464	20643	0	1632							
	PAP-213	585	38934	0	571	0	0	8197	425	778000	267000	286
	SART2-93	121	282	0	0							
	MRP3-1293	98	415	0	186							
14	C-35	121	181	0	425							
	SART3-302	1044	5609	0	0	0	2398	719	2905	415	4722	255
	Lck-246	53	47	0	0							
	SART3-309	48	43	0	0							
15	C-35	23944	24099	0	541							
	SART3-109	2522	2734	0	0	0	0	82640	387000	23737	65652	85
	Lck-488	189	280	0	0							
	SART2-93	143	5751	0	0							
16	C-35	22269	21464	0	0							
	SART2-93	58	53	0	0	2767	143	87150	403900	52	156	100
	Lck-486	56	49	0	0							
	EGF-R-800	54	57	0	0							
17	C-35	14683	14881	0	0							
	Lck-488	3149	2914	0	311	0	0	10	9	29000	99300	128
	SART2-161	1912	1573	0	0							

	Lck-486	1340	1143	0	0							
18	C-35	15844	16666	0	399							
	SART3-302	3177	24118	0	0	722	1730	616	1779	12	39	129
	Lck-246	1341	1070	0	0							
	UBE2V-43	107	15917	0	0							
19	C-35	23316	23405	0	1506							
	HNRPL-140	398	332	0	0	5167	9082	104	184	51305	303630	155
	WHSC2-103	295	238	0	0							
	SART3-302	211	206	0	0							
20	C-35	19281	18608	0	0	0	680	49	106	3300	8940	300
	WHSC2-103	73	39	0	0							
21	C-35	15075	14541	185	0							
	PSA-248	7774	20174	0	0	0	192	38	34	77	972	345
	SART3-109	7677	12448	0	229							
	SART2-93	439	565	0	0							
22	C-35	15237	NA	0	NA							
	MRP3-1293	201	NA	0	NA	0	NA	498	NA	294280	NA	56
	PAP-213	151	NA	0	NA							
	SART2-93	91	NA	0	NA							
23	C-35	7638	7156	0	193							
	Lck-488	4385	4409	0	163	0	0	2256	7040	33300	73100	145
	MRP3-1293	1712	3065	0	0							
	SART2-93	1009	863	0	0							
24	C-35	25860	25691	0	0							
	SART3-302	16190	14802	0	0	1911	2846	14	10	27900	207	154
	HNRPL-501	51	703	0	745							
	WHSC2-103	48	78	0	495							
25	C-35	8775	15107	0	92							
	SART3-109	11666	11650	0	0	0	0	225	796	3101	26466	164
	Lck-486	2430	2362	0	0							
	SART2-93	199	8252	0	685							
26	C-35	12393	9983	0	71	340	470	1880	2880	972	2540	237
	SART3-734	1336	1491	0	0							
27	C-35	1585	1312	0	0							
	Lck-246	378	292	0	0	2087	1007	254	281	53	22	209
	ppMAPkkk-432	134	110	0	0							

	WHSC2-103	122	113	0	0							
28	C-35	36436	36269	0	0							
	SART3-511	488	529	80	0	0	0	48	36	437	835	481[e]
	SART3-734	132	185	0	0							
29	C-35	37661	NA	0	NA							
	SART3-109	1130	NA	0	NA	0	NA	10	NA	3950	NA	58
	Lck-488	312	NA	0	NA							
	SART2-93	251	NA	0	NA							
30	C-35	37657	40695	0	364							
	SART2-93	173	285	0	0	869	1401	5413	8253	248	179	483[e]
	SART3-109	162	1713	0	0							
	Lck-488	101	249	0	0							
31	C-35	20059	19527	0	1288							
	WHSC2-141	44	63	0	0	0	0	4636	9313	17168	56666	245
	CypB-129	41	41	0	0							
	Lck-246	38	40	0	0							
32	C-35	42340	42650	0	1132							
	Lck-488	259	440	0	105	3396	2213	544	990	21749	2394	186
	PAP-213	209	48434	0	490							
	SART2-93	186	398	0	268							
33	C-35	26949	26521	0	0							
	MRP3-1293	192	86	0	0	0	0	9	5	82250	145755	69
	SART2-93	107	108	0	0							
	PSA-248	86	149	0	0							
34	C-35	38919	38897	0	0							
	ppMAPkkk-432	721	765	0	0	2602	3790	1476	1288	162110	85920	517[e]
	Lck-246	248	281	0	0							
	HNRPL-140	248	226	0	0							
35	C-35	32171	32810	0	236							
	Lck-90	359	367	0	0	0	0	28	22	11	8	374
	Lck-449	173	164	0	1090							
	SART3-734	154	152	0	0							
36	C-35	17909	28156	0	901							
	PAP-213	388	19613	0	710	1795	3994	53	746	11	14	324
	SART2-93	479	445	0	466							
	PSMA-624	223	1039	0	0							

37	C-35	38207	38310	0	0	0	0	15	28	236	631	184
	Lck-488	185	130	0	0							
	Lck-486	122	85	0	0							
	SART2-93	114	101	0	0							
38	C-35	36429	35672	0	567	3527	6364	133	80	136	143	168[e]
	Lck-488	3059	3572	0	0							
	SART2-93	794	295	0	997							
	PSA-248	559	3464	0	0							
39	C-35	87	493	0	0	0	323	36	243	118	1020	245[e]
	PSA-248	541	10891	0	0							
	Lck-488	95	112	0	0							
	SART2-93	72	86	0	826							
40	C-35	37761	37889	44	0	0	2350	4	7	2070	7510	189
	SART2-93	145	194	0	0							
	Lck-488	131	212	0	0							
	PSA-248	85	1029	0	0							
41	C-35	24642	NA	0	NA	0	NA	103000	NA	14500	NA	117
	SART2-93	340	NA	0	NA							
	Lck-488	175	NA	0	NA							
	MRP3-1293	151	NA	0	NA							
42	C-35	22949	NA	0	NA	0	NA	898	NA	171	NA	77[e]
	Lck-488	337	NA	0	NA							
	SART2-93	309	NA	0	NA							
	PSA-248	261	NA	0	NA							

Abbreviations: CTL, cytotoxic T lymphocytes; AFP, α-fetoprotein; DCP, des-γ-carboxy prothrombin, OS, overall survival; NA, not assessed

[a]Antigen peptides used for vaccination.

[b]Values indicate the fluorescence intensity unit (FIU) of plasma IgG reactive with the vaccine peptides before and after vaccination. The augmented IgG responses after vaccination are underlined.

[c]Values indicate the number of spots per 10^5 peripheral blood mononuclear cells (PBMCs) reactive with the vaccine peptides or CEF peptides in IFN-γ ELISPOT assay before and after vaccination. The augmented T cell responses after vaccination are underlined.

[d]Tumor markers were measured before and after vaccination. The decreased values after vaccination are underlined.

[e]Patients were alive at the last follow-up.

Supplementary Table 3. Humoral immune repsonses (IgG subclass)

Patient No.	Peptide[a]	IgG1 response[b]		IgG2 response[b]		IgG3 response[b]		IgG4 response[b]	
		Before	After	Before	After	Before	After	Before	After
1	C-35	14542	14553	0	0	22	21	38	38
	Lck-208	12	0	0	0	0	0	0	0
	PAP-213	0	33	0	0	0	0	0	0
	PTHrP-102	0	0	26	27	10	0	0	0
2	C-35	23430	22955	787	594	48	43	49	40
	Lck-208	0	0	0	0	0	0	0	0
	PAP-213	20	21	10	20	14	18	0	0
	PTHrP-102	0	0	0	0	0	0	0	0
3	C-35	22238	23946	0	14669	0	10579	50	230
	Lck-246	34	14012	125	2216	22	2802	11	115
	CypB-129	840	4836	0	0	61	89	13	15
	WHSC2-141	56	603	69	106	151	170	51	51
4	C-35	68	231	0	0	0	32	0	0
	Lck-208	109	111	26	54	21	24	0	18
	PSMA-624	34	48	81	1243	0	0	0	16
	PAP-213	33	23399	11	2390	0	7902	0	130
5	C-35	22912	22836	112	368	133	305	60	118
	CypB-129	132	119	26	29	29	23	0	0
	UBE2V-43	54	49	25	22	15	13	0	0
	HNRPL-140	19	16	15	20	74	63	0	0
6	C-35	NA	NA	NA	NA	NA	NA	NA	NA
	HNRPL-501	NA	NA	NA	NA	NA	NA	NA	NA
	ppMAPkkk-432	NA	NA	NA	NA	NA	NA	NA	NA
	WHSC2-103	NA	NA	NA	NA	NA	NA	NA	NA
7	C-35	2882	2239	0	0	11	0	0	0
	MRP3-1293	28	42	21	21	40	38	17	18
	Lck-488	73	64	43	37	124	104	33	25
	SART2-93	30	89	43	65	71	71	30	31

8	C-35	20625	22054	0	0	21	24	19	18
	SART2-93	61	63	73	92	57	60	103	101
	Lck-488	87	2164	77	104	73	96	70	67
	MRP3-1293	44	48	14	24	45	41	0	0
9	C-35	9528	18602	0	234	0	104	0	0
	UBE2V-85	3865	8973	22	78	0	69	0	0
	Lck-422	4260	2754	0	0	0	0	0	0
	UBE2V-43	1594	22835	0	3062	0	2303	0	99
10	C-35	395	2344	0	73	0	895	0	0
	PAP-213	582	21398	32	1120	14	78	0	385
	Lck-486	94	8608	34	271	81	7213	12	102
	Lck-488	89	465	85	82	101	104	35	23
11	C-35	NA	NA	NA	NA	NA	NA	NA	NA
	WHSC2-103	NA	NA	NA	NA	NA	NA	NA	NA
	ppMAPkkk-432	NA	NA	NA	NA	NA	NA	NA	NA
	WHSC2-141	NA	NA	NA	NA	NA	NA	NA	NA
12	C-35	10211	9397	555	634	610	2863	0	0
	SART3-109	654	584	243	114	3052	2459	0	0
	Lck-90	68	101	88	98	40	47	0	10
	HNRPL-501	46	636	61	83	0	57	0	0
13	C-35	4357	15661	32	191	57	277	0	0
	PAP-213	13	23844	480	12524	0	14805	0	247
	SART2-93	22	139	60	66	21	99	0	0
	MRP3-1293	0	118	0	18	0	0	0	0
14	C-35	51	166	0	0	0	0	0	0
	SART3-302	741	4726	103	116	0	0	0	0
	Lck-246	70	54	73	58	0	24	20	19
	SART3-309	18	15	52	43	0	13	0	0
15	C-35	22920	23069	47	40	0	17	0	0
	SART3-109	1016	1372	27	29	12	22	0	0
	Lck-488	115	179	116	131	82	135	322	453

	SART2-93	63	6405	107	644	32	135	232	427
16	C-35	17846	16162	17	12	17	17	62	50
	SART2-93	36	38	76	68	18	22	0	0
	Lck-486	19	23	61	65	0	13	0	0
	EGF-R-800	39	46	30	35	0	0	0	0
17	C-35	7234	6305	117	101	0	16	0	0
	Lck-488	70	102	1455	1187	36	33	24	19
	SART2-161	0	14	1097	793	0	0	0	0
	Lck-486	16	54	1287	969	0	15	0	0
18	C-35	9553	13568	0	26	0	15	0	0
	SART3-302	3476	23874	13	3220	0	199	0	77
	Lck-246	88	130	137	140	80	71	31	24
	UBE2V-43	104	23857	0	15539	0	1031	0	109
19	C-35	23874	23940	583	3016	263	353	33	2551
	HNRPL-140	0	0	0	0	0	0	0	0
	WHSC2-103	0	0	0	0	17	16	15	13
	SART3-302	0	0	0	0	0	13	0	0
20	C-35	0	12211	0	0	0	0	0	0
	WHSC2-103	0	61	40	26	17	0	12	0
21	C-35	17924	18036	45	53	11	15	0	0
	PSA-248	9124	24199	41	157	39	652	15	56
	SART3-109	2527	8589	3889	6330	23	100	24	31
	SART2-93	68	71	413	453	51	52	26	21
22	C-35	NA	NA	NA	NA	NA	NA	NA	NA
	MRP3-1293	NA	NA	NA	NA	NA	NA	NA	NA
	PAP-213	NA	NA	NA	NA	NA	NA	NA	NA
	SART2-93	NA	NA	NA	NA	NA	NA	NA	NA
23	C-35	3376	3192	0	0	0	0	81	92
	Lck-488	5049	5073	79	93	114	134	0	0
	MRP3-1293	14	42	718	1405	0	0	0	0
	SART2-93	44	50	665	607	22	27	11	12

24	C-35	24030	24211	1842	2668	129	187	30	42
	SART3-302	20448	20872	104	184	110	152	14	22
	HNRPL-501	176	409	59	69	81	189	32	48
	WHSC2-103	19	19	56	61	30	49	0	0
25	C-35	6393	11395	0	176	20	91	0	31
	SART3-109	4975	5071	34	55	4093	3865	0	0
	Lck-486	42	56	0	0	553	587	0	0
	SART2-93	57	7873	127	3982	90	148	120	142
26	C-35	5764	3906	0	0	18	14	0	0
	SART3-734	735	873	96	120	65	35	25	11
27	C-35	440	465	29	30	0	10	0	0
	Lck-246	54	61	91	91	41	42	0	11
	ppMAPkkk-432	12	11	36	33	0	0	0	0
	WHSC2-103	23	27	30	29	83	87	0	0
28	C-35	21423	16081	0	0	13	199	11	0
	SART3-511	211	142	30	33	23	21	11	0
	SART3-734	180	147	13	0	85	54	26	20
29	C-35	NA	NA	NA	NA	NA	NA	NA	NA
	SART3-109	NA	NA	NA	NA	NA	NA	NA	NA
	Lck-488	NA	NA	NA	NA	NA	NA	NA	NA
	SART2-93	NA	NA	NA	NA	NA	NA	NA	NA
30	C-35	23354	24117	11	3017	238	7071	73	894
	SART2-93	55	66	89	100	57	66	52	49
	SART3-109	79	1299	0	35	0	38	0	0
	Lck-488	84	146	108	115	78	76	74	53
31	C-35	11578	12255	30	15	12	0	0	0
	WHSC2-141	0	0	20	42	20	63	0	11
	CypB-129	0	0	0	0	0	0	0	0
	Lck-246	0	0	0	0	0	0	0	0
32	C-35	23986	24058	5701	6048	143	158	40	43
	Lck-488	61	204	73	87	75	98	15	28

	PAP-213	50	23097	41	1404	19	4307	0	64
	SART2-93	49	170	76	66	45	83	21	44
33	C-35	12735	11094	0	0	0	0	0	0
	MRP3-1293	131	57	13	0	19	23	74	72
	SART2-93	36	29	71	71	80	69	51	43
	PSA-248	23	29	0	0	0	0	0	0
34	C-35	23382	23208	1072	1073	23573	23635	100	83
	ppMAPkkk-432	0	0	0	13	162	149	0	0
	Lck-246	42	55	57	48	40	47	19	17
	HNRPL-140	131	137	27	32	11	13	0	0
35	C-35	18120	17355	0	0	0	0	12	14
	Lck-90	486	368	79	85	47	33	31	18
	Lck-449	45	40	237	271	45	54	31	20
	SART3-734	28	26	177	216	47	45	30	20
36	C-35	12233	20374	71	149	21	624	0	0
	PAP-213	306	24592	18	18041	14	10662	0	184
	SART2-93	652	691	80	86	59	58	20	20
	PSMA-624	45	851	11	15	147	180	11	11
37	C-35	24640	24636	121	446	2151	2569	33	98
	Lck-488	68	54	58	69	286	220	0	0
	Lck-486	17	16	0	0	31	27	0	0
	SART2-93	24	29	62	58	66	60	0	0
38	C-35	22248	18149	0	15	106	27	431	77
	Lck-488	4481	4064	133	217	653	687	90	45
	SART2-93	0	127	25	110	94	83	14	0
	PSA-248	0	1835	0	30	19	35	0	0
39	C-35	0	230	0	0	0	0	0	17
	PSA-248	254	10053	0	95	0	0	0	0
	Lck-488	42	56	100	88	43	45	16	38
	SART2-93	33	39	72	79	35	38	20	46
40	C-35	24215	24183	23824	23443	178	143	146	145

	SART2-93	43	48	90	74	52	50	14	15
	Lck-488	69	94	111	119	116	110	27	26
	PSA-248	0	26	0	0	0	368	0	0
41	C-35	NA	NA	NA	NA	NA	NA	NA	NA
	SART2-93	NA	NA	NA	NA	NA	NA	NA	NA
	Lck-488	NA	NA	NA	NA	NA	NA	NA	NA
	MRP3-1293	NA	NA	NA	NA	NA	NA	NA	NA
42	C-35	NA	NA	NA	NA	NA	NA	NA	NA
	Lck-488	NA	NA	NA	NA	NA	NA	NA	NA
	SART2-93	NA	NA	NA	NA	NA	NA	NA	NA
	PSA-248	NA	NA	NA	NA	NA	NA	NA	NA

Abbreviation: NA, not assessed

[a]Antigen peptides used for vaccination.

[b]Values indicate the fluorescence intensity unit (FIU) of plasma IgG subclasses reactive with the vaccine peptides before and after vaccination. The augmented IgG responses after vaccination are underlined.
